# Psychedelics reopening windows of development in mice

**DOI:** 10.1038/s44271-023-00012-1

**Published:** 2023-09-05

**Authors:** Antonia Eisenkoeck

**Affiliations:** Communications Psychology, https://www.nature.com/commspsychol/

## Abstract

A new study in *Nature* shows psychedelics’ ability to reopen a critical period of development in mice. This shared property across psychedelic drugs was proportional to the duration of acute subjective effects of the drugs in humans.

**Figure 1:**
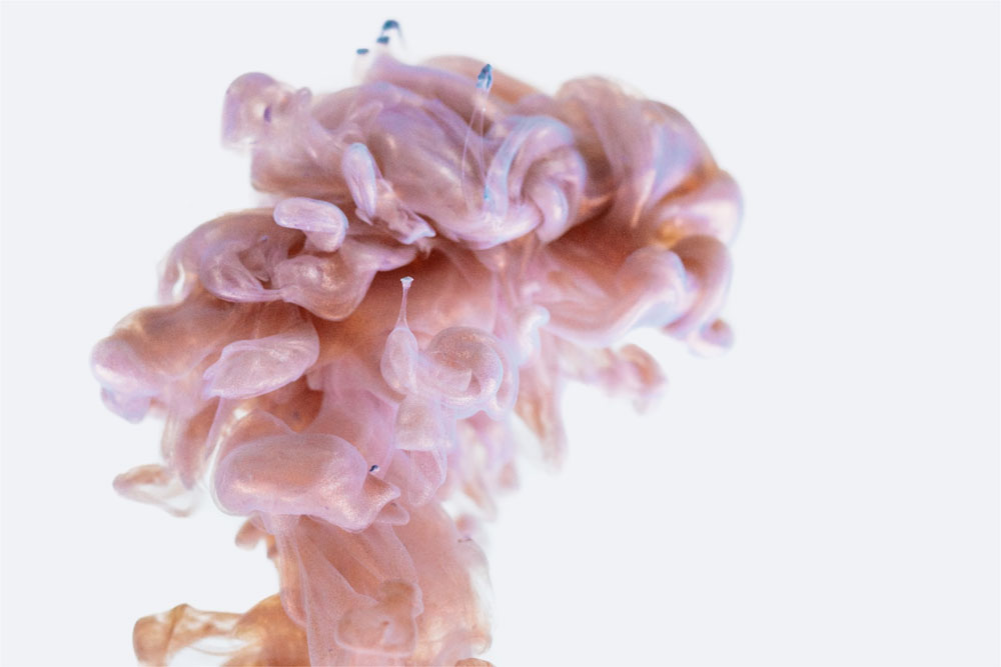
Pawel Czerwinski on unsplash.com

Psychedelics are a group of drugs associated with altered states of consciousness. They have been used for therapeutic and spiritual purposes by various cultures for thousands of years. Recently, psychedelics have received renewed interest, not least because of their potential application in treatment for mental health conditions such as depression, addiction or PTSD.

But psychedelic drugs vary greatly in their chemical properties, which raises the question: do effects of these drugs on the brain rely on the same mechanism?

Romain Nardou and colleagues at Johns Hopkins University addressed this knowledge gap  by studying the effects of psychedelics on social behaviour in mice. Mice form positive associations with socializing—but only during a critical period in adolescence. Nardou and team found that adult mice treated with the psychedelics LSD, psilocybin, MDMA or ketamine found social experiences more rewarding than mice treated with saline or cocaine^1^. This suggests a unifying mechanism of psychedelics reinstating the youthful critical period—an effect which was proportional to the respective drug’s duration of acute subjective effects in humans. Neurons in the brains of mice treated with psychedelics had become more sensitive to the hormone oxytocin, which is further evidence for an induced ‘metaplasticity’ assumed to underlie the establishment of critical periods.

This novel conceptual framework of the underlying mechanisms of psychedelics, opens up avenues for future research and discourse. If research shows that it is applicable to humans, this will have relevance for clinical practice, in which a nuanced understanding of brain plasticity and its ability to positively change cognition and behaviour is key.
